# Introducing the Language of “Relativity” for New Scaffold Categorization

**DOI:** 10.3390/bioengineering6010020

**Published:** 2019-02-26

**Authors:** Haobo Yuan

**Affiliations:** School of Engineering, University of South Australia; Mawson Lakes Blvd, Salisbury 5095, Australia; Haobo.yuan@mymail.unisa.edu.au; Tel.: +61-08-8302-6611

**Keywords:** language of relativity, scaffold categorization, evolution of scaffold, seven-folder logics, cell culture, 3D scaffold, dynamicity and dimensionality, traditional scaffold, novel scaffold, future scaffold engineering, laws of system evolution, 3DPVS, vibrating nature of universe.

## Abstract

Research related with scaffold engineering tends to be cross-domain and miscellaneous. Several realms may need to be focused simultaneously, including biomedicine for cell culture and 3D scaffold, physics for dynamics, manufacturing for technologies like 3D printing, chemistry for material composition, as well as architecture for scaffold’s geometric control. As a result, researchers with different backgrounds sometimes could have different understanding towards the product described as ‘Scaffold’. After reviewing the literature, numerous studies termed their developed scaffold as ‘novel’, compared with scaffolds previously designed by others using comparing criterion like ‘research time’, ‘manufacturing method’, ‘geometry’, and so on. While it may have been convenient a decade ago to, for example, categorize scaffold with ‘Dualistic Thinking’ logic into ‘simple-complicated’ or ‘traditional-novel’, this method for categorizing ‘novelty’ and distinguishing scaffold is insufficiently persuasive and precise when it comes to modern or future scaffold. From this departure of philosophical language, namely the language of ‘relativity’, it is important to distinguish between different scaffolds. Other than attempting to avoid ambiguity in perceiving scaffold, this language also provides clarity regarding the ‘evolution stage’ where the focused scaffolds currently stand, where they have been developed, and where in future they could possibly evolve.

## 1. Introduction

Cell culture scaffold is defined as a class of artificially-created biomedical products used for culturing cells in vitro, through mimicking some real tissue properties. Scaffold engineering, in this connection, has developed in two chief avenues. One can be ‘static into dynamic’, with proven effects that dynamic cultures have benefits over static ones. In this direction, scaffolds were focused on dimensional difference, so to speak, ‘2D into 3D’. In addition, artificially created 3D scaffolds, which have the nature of being passive (i.e., ‘static’), have been utilized, helping external culturing mimic real tissue in 3D environments with better performance, compared with traditional 2D cell culturing methods. In another direction, attempts have been made to develop cell culturing with some dynamic properties or possibly combine scaffold with some mechanical means like shakers, both of which were aimed to potentially approximate part of dynamic environments in real tissue. Provided that the new categorization of scaffold, which uses the ‘evolution’ criteria as the ‘center of gravity’, can be studied in two ways, namely the evolutional ‘points’ in dimensionality as well as in dynamicity. A ‘point’ could represent a certain combination of properties, which is organized in a definite time and place, and fulfills a definite function in one system or another. Take scaffold for instance: a ‘point of the scaffold’ can be designated through the number of central or predominating properties inside the scaffold. The philosophy will be more easily perceived when it comes to the seven-layer classifying process.

On other hand, this paper aims to introduce the language of ‘relativity’ and apply it as the novel scaffold categorization method. Currently, existing scaffolds in biomedical worlds will be defined as the scaffold No. 1, 2, 3, and 4. In this connection, each scaffold will be introduced and studied briefly. Then, the contents of each will be organized in ‘summary’ format, instead of a detailed ‘review’ or explanation. Future scaffold No. 5, which consists a scaffold of ‘self-integration’, will be introduced, with potential 3DPVS (3D Printed Vibratory Scaffold) as one essential part which was justified via studying scaffold No. 1, 2, and 3, as well as simultaneously investigating ‘Laws of System Evolution (LSE)’ into scaffold engineering. It is worth noting that the scaffold No. 5 concept includes a multiple-aspect justification and has been conducted previously by author’s research group and transmitted into publications [1,2]. Scaffold No. 6 and 7 are scaffolds that might potentially appear in relatively long-term future, after the mature stage of scaffold No. 5, which will be briefly discussed later. Scaffold No. 1 to No. 7 will compose the full seven-layer evolutional ladder of scaffold engineering. Besides the categorizations, a possible further sub-classifying, possibly existing under each of the seven hierarchies, will also be briefly mentioned, which might be interesting to follow-up with in the future. 

## 2. Introducing the Language of ‘Relativity’

‘Relativity’ is a complicated system that has been applicated in both philosophy and modern physics [3]. To understand ‘relativity’, we use the terms ‘novel’ and ‘traditional’ regarding scaffold as examples. From the current review of literature, the term ‘3D scaffold’ predominantly refers to 3D passive or static scaffold. ‘Advanced 3D scaffold’ or ‘novel 3D scaffold’ refers to a number of 3D scaffolds fabricated with more sophisticated material complex and higher controlled geometrics. However, these ‘traditional 3D scaffolds’ can be and have been termed as ‘novel, advanced, or innovative’ by researchers when compared with earlier 2D culture plates, platforms, substrates, or scaffolds, which literally exist as more traditional. Therefore, the term ‘novel’ can only be established based on ‘relativity’. Misunderstanding easily occurs when using a term like ‘novel cell culture scaffold’ without a permanent reference object or centre and this indeed becomes a limitation for researches to communicate ‘novelty’ inside scaffold. 

As philosophy states, exact language is necessary for exact understanding [3]. In this paper, we made efforts to define scaffold based on ‘relativity’ that could make it possible to know what is being discussed, from which point of view, and in what connection. The fundamental property of the new definition is that all concepts of ‘scaffolds’, as well as their mutual relationships, are concentrated around one idea. This idea is about ‘evolution’, namely the cell culture scaffold’s evolution. Seven levels of scaffolds have been defined to potentially illustrate the whole evolution process of scaffold engineering, which covers the past, present, and future. With this betterment in scaffold’s categorization, future scaffold researchers might be benefited, howsoever small, with the increased clarity, precision, and understanding during their studies. 

## 3. The Novel Seven-Layer Scaffold Categorization

Using the language of ‘relativity’, scaffolds will be categorized in a seven-layer format, which helps indicate the relative position of each scaffold or each group of scaffolds that share some common traits inside the whole scaffold’s evolutional process. Previous and major current scaffolds would be defined as scaffold No. 1, 2, and 3; future 3D Vibratory Scaffold, which will be developed via the current research period, would be termed as scaffold 5, and the bridging scaffold products between scaffold No. 1, 2, 3, and 5, would be referred to as scaffold 4. Scaffold No. 7 refers to the ideal scaffold ultimately evolved from scaffold No. 5; this evolution could practically occur in the future, but is limited by existing technology in bioscience and materials, as well as the immature development of scaffold No. 5. Scaffold bridging the scaffold No. 5 and 7 can be named as scaffold No. 6. As a matter of fact, scaffold No. 6 and 7 could merely be predicted and perceived theoretically, but much work in this direction will be needed for future researchers. The seven-fold scaffold classification will be discussed in three groups: scaffold No. 1, 2 and 3; scaffold No. 4 and 5; scaffold No. 6 and 7; and a concluding comparison of the seven levels of scaffold is illustrated in Table 1. 

### 3.1. Scaffold Number One, Two, and Three

In this section, three scaffold classes will be introduced. Scaffold No.1, 2, and 3 constitute the predominantly major part of currently existing literature on scaffold engineering. 

#### 3.1.1. Scaffold No. 1

Scaffold No. 1 is the traditional 2D static scaffolds used for 2D cell cultures. Scaffold No. 1 is the product initiating the evolution process of scaffold engineering. Invented more than 50 years, 2D scaffolds or platforms are sometimes still used by researchers due to the inexpensive and easy-fabricating properties [4,5], though many of them have been gradually replaced by 3D scaffold, which starts as scaffold No. 2. For both dimensionality and functionality, scaffolds in this category stay at the bottom of the scaffold’s evolutional ladder. This scaffold product used to be considered ‘novel’ when comparing it with ‘traditional’ 2D plastic plates or containers used in much early 2D cell culturing. The 2D substrates remain as flat surfaces with dimensions in the z-axis at a hundred nanometre level where cell growth is predominantly controlled in an x-, y-direction [5], e.g., alignment of cells along shallow grooves. 

#### 3.1.2. Scaffold No. 2

Scaffold No. 2 is the early-phase 2.5D or 3D static or passive scaffolds used for 3D cell culture; these scaffolds occupy much of the early literature concerning ‘3D cell culture’ and they are usually designed with single and simple material composition and geometries. Scaffold No. 1 into No. 2 follows the evolution law of ‘lower dimensional into higher dimensional’. If it is assumed that scaffold in this category is ‘novel’, then the comparison becomes reasonable only when the compared object is scaffold No. 1, which stays at a relatively lower ladder or the less developed scaffold No. 2 in same ladder. Scaffold No. 2 could also be considered as the early-stage or immature products of 3D scaffold, which will be defined as scaffold No. 3. Work of scaffold No. 2 concentres around mid-20th century, where natural extracellular matrixs (ECM) or gels composed of natural or synthetic polymers were used [6,7]. Conclusion of culturing cell on flat 2D versus 3D can significantly alter cellular responses generally originated from such approaches.

#### 3.1.3. Scaffold No. 3

Scaffold No. 3 is the currently developed or to-be-developed 3D static scaffold used for advanced cell culture applications; these scaffolds tend to have multiple material composition, multi-functional biomedical control, and complicated architectural structures. This scaffold is also the major focus of researchers in the current scaffold engineering field. When the term ‘3D scaffold’ has been used in past years, it mostly referred to scaffold No. 3. This scaffold group chiefly follows the law of ‘simple structure and composition into a complicated and complex one’. Scaffold No. 3 includes the major part of scaffolds termed as novel or advanced by recent researchers. This subconscious belief could lie on the fact that scaffold No. 3 is surely ‘novel’ over scaffolds No. 1 and 2, thus leading people to take it for granted that the ‘novel’ scaffold is equivalent as scaffold No. 3. However, such conception is not true, according to the ‘relativity’ philosophy discussed earlier; if researcher were to work on scaffold No. 4 and 5 as their project ‘novel scaffold design’, then all scaffold No. 1, 2, and 3 would be logically categorized into a ‘traditional’ category, which seems to be scientific and provides with better clarity and understandability to other researchers. 

The appearance of scaffold No. 3 could be partly due to the invention of 3D printing as the third industrial revolution [8,9], which flourished in tissue engineering. Opposed to 3D scaffolding brings about the concept of scaffold-free TE (tissue engineering), which, based on the assembly of building ‘cell sheets’ and ‘spheroids’ blocks, provides merits over conventional scaffolds No. 1 and 2, especially regarding tissue formation efficiency. However, it cannot replace the role of 3D scaffold, which provides a biomimetic environment that delivers or controls the release of growth and differentiation factors as well as the robust structure protecting cells from possible damage via external factors [10]. Therefore, if scaffold-free is considered, it could generally rate at evolutional ladder at scaffold No. 3 or perhaps No. 4, if a more advanced dynamic or dimensional properties are embedded within.

### 3.2. Scaffold Number Four and Five

This section will discuss two scaffold categorizations: scaffold No. 4 and scaffold No. 5. Future scaffold research starts from here. 

#### 3.2.1. Scaffold No. 4

Scaffold No. 4 is the scaffold with either some dynamic, partially dynamic, or active to some extent properties. Current 3D scaffolds are fabricated with shape-changing materials and can be the typical products in this category. Bridging traditional scaffold No. 1, 2, and 3, and future novel scaffold No. 5, aspects regarding scaffold No. 4 would occupy a significant positionality towards developing new scaffolds. A chief feature of scaffold No. 4 is its increasingly integrated dynamic functionality inside a 3D scaffold. Compared with scaffold No. 3, material composition would be the major difference between both. New materials, for instance smart materials, which can generate some movable or changeable actions of scaffold under specific stimulus [11,12], could be utilized experimentally on scaffold engineering. Relation of scaffold No. 4 to No. 5 is similar as the relation of scaffold No. 2 to No. 3. Further, scaffold No. 4 could be considered as the immature or preparing-stage product of scaffold No 5, whereas the dynamic properties not integrated into scaffolds as an indivisible unit or the aimed dynamicity can only partly be fulfilled by scaffold. In brief, when evolutional ‘points’ of dynamicity is concerned, ‘traditional scaffold’ starts to indicate the whole categories of scaffold No. 1, 2, and 3, which has the nature of being passive or static. In this connection, scaffold No. 4 and No. 5 could be scientifically defined as ‘novel or advanced’. 

In terms of the currently emerged organ-printed product or bio-printing that has a similar function as scaffold, its level of ideality might be seen as a corresponding level of scaffold 4, based on the level of advance in dimensional control and in dynamic or vibratory properties. Since bio-printing is in infancy [9,13], further development may make it evolve as a No. 5 level product. An interesting evolutionary indication of vibration on a bio-printed product or scaffold is whether or not it could controllably mimic human-like cellular vibrating in definite cell culture and to what extent it can mimic. Other scaffold products, such as scaffolds with advanced bio-restorability, bio-activity, and timely-changing properties, could be positioned at scaffold No. 4 level with similar logic. Furthermore, the use of a decellularized extracellular matrix (ECM) of a tissue is another promising trend, part of which would possibly stand out as evolutionary, similar as scaffold No. 4, considering its beneficial advances in bio-dynamicity and real tissue structuring. Moreover, the advent of whole organ decellularization brings extracellular matrix scaffolds suitable for organ engineering [14,15,16]. Current preliminary works in this direction can be considered at the No. 4 level and future scaffolds have the potential to arrive at a No. 5 or greater.

#### 3.2.2. Scaffold No. 5

Scaffold No. 5 is the future 3D dynamicity-integrated Scaffold that usually unifies one chief dynamic from external to internal, which is an unattainable standard for current scaffolds. In brief, scaffold No. 5 is the scaffold of ‘self-integration’, for it is a scaffold that has reached ‘unity’. Evolved from scaffold No. 1, 2, 3, and 4, it changes its role of being static and passive into active and dynamic. Unification of separate parts—e.g., making an external dynamic device and 3D scaffold one whole unit—is the chief feature of scaffold No. 5. To be more specific, scaffolds No. 1, 2, and 3 tend to be limited in receiving vibrations through connecting culturing platforms to external vibrators or mechanical shaker vibrators [17,18], while scaffold No. 5 is the 3D vibratory scaffold capable of generating proper vibrations, which are required by specific cell cultures in an internal and integrated manner. Such an approach would mitigate several limitations in traditional dynamic cell cultures [2]. A novel future scaffold concept, 3DPVS (3D printed vibratory scaffold) [1], might be a typical representative in the scaffold No. 5 category, which could even be potentially equivalent to scaffold No. 5. This is based on the current limitations of scaffold as studied [2], in addition to the future pointing direction, evaluated from the ‘laws of system evolution’ [1,19,20]. 

Conceptual 3DPVS is generated as a byproduct of No. 5, which concerns 3D printed technology and vibration that transforms the passive role of scaffold receiving vibration into an actively generating vibration. It would adequately include merits and properties of traditional scaffold No. 2 and 3, as well as the benefits of exact vibratory functions onto cell culture. Vibration compared with other dynamic properties in cell culture has been studied as an optimal dynamicity to be applied on scaffold No. 5. Moreover, novel 3D printing (3DP) has been the technology that bridges traditional scaffold No. 1, 2, and 3 to fabricating novel 3D vibratory scaffold. In connection with scaffold No. 5, scaffold No. 4 could logically include the group of scaffolds under testing that experiment with different vibration mechanisms and different materials. Thus, No. 4 could gradually approximate yet remain distant from the goal of scaffold No. 5. In other words, the basic logic behind scaffold No. 5 is ‘static into dynamic’, ‘passive into active’, and ‘separated into integrated’. ‘Vibration’ inside scaffold No. 5 will be much finer and subtler compared with the ‘vibration’, as applied in previous scaffolds. This is perhaps a result of a molecular vibrating level. Furthermore, better applications for in vitro cell studies might be another character for scaffold No. 5. As a result, it is reasonable to predict that scaffold No. 5 would play a promising role in the near future of scaffold engineering. 

### 3.3. Scaffold Number Six and Seven

Scaffold No. 6 and No. 7 refer to scaffolds in the relativley long-term future. They might start playing roles in future engineering when the progress of scaffold No. 5 becomes solid, applicable, and mature. At the current stage, their value might predominately be considered as conceptual and theoretical. 

#### 3.3.1. Scaffold No. 6

Scaffold No. 6 is the intermediate or bridging scaffold between scaffold No. 5 and No. 7; its related role towards scaffold No. 7 is like that of scaffold No. 4 towards No. 5. At its current stage, scaffold No. 6 and 7 remains theoretical and might not appear in the near future until research regarding scaffold No. 5 has been solid and successful. In terms of potential properties, scaffold No. 6 might open the avenue of multiple external dynamic properties, where functions in cell culture could merge with the scaffold itself. Multi-dynamicity plus integration would be the typical feature for scaffold No. 6, which evolves from scaffold No. 5. No. 6 focuses on single-dynamicity that integrates into the vibration property in 3DPVS. Compared with ‘self-integration’ in scaffold No. 5, scaffold No.6 possibly attains the capability for ‘multipleness-integration’. Developed from 3D, the dimensionality of scaffold No. 6 might be expanded, provided that realistic 4D (4-dimensional) engineering could come into its application. Standing relatively close to scaffold No. 7, as well as following same evolutional law, scaffold No. 6 might cease to be artificial as external cell culture scaffold and becomes more bio-mimic to real organs or tissues. To conclude, scaffold No. 6 could be the future direction following the development and innovation of scaffold No. 5. A successful design and mature application would open avenues for scaffold No. 6. 

On other hand, past-to-present scaffolds or bio-products witnessed three generations of biomaterials, from common and borrowed materials to engineered implants, as well as to bioengineered implants using bioengineered materials. The revolution of materials also affects the revolution of scaffolds. Although it is not clear what the following evolution of material indicates, it could very possibly contribute to the emerge of products as scaffold No. 6. 

#### 3.3.2. Scaffold No. 7

Scaffold No. 7 is the ‘ideal’ future scaffold, as it is highly dimensional and dynamic, and is defined as a scaffold capable of fully mimicking, replacing, or manipulating the in vivo cell culture of 3D microenvironments. Scaffold No. 7 would reach its full development that possesses ‘everything’ a scaffold can possess. It might close the gap of cell culture between the external and internal. One chief evolutional point of Scaffold No. 7 dwells at dynamicity, i.e., more dynamic functions or properties beyond ‘vibration’, which is the chief feature of scaffold No 5, will be endowed. Thus, scaffold No. 7 will probably be the ultimate 3D or higher-dimensional dynamic scaffold, as far as the currently perceivable properties of scaffold are concerned. Above which the concept of 3D scaffold might cease to exist and further evolution ladder would become unpredictable. A scaffold No. 7 might also be viewed like perceiving current mechanical robots and the future artificial-intelligence resulted human-mimic robot, the latter of which has only been shown in science fiction. In short, scaffold No. 7 might be the perfect scaffold. 

Further discussions of ‘vibration’ and ‘dimensionality’, from an evolutional point of view, substances the that universe consists of vibrations and matter, or of matter in a state of vibration, or of vibrating matter. The rate of vibration is an inverse ratio to matter’s density. Higher and finer vibration generally correlates to a higher consciousness of definite objects or creatures, while low-level vibrations are connected with more mechanicalness. We could understand this from the difference between living creatures and ‘dead’ artificial machines. Moreover, high dynamicity could mean a higher level of vibration in anobject, whether predictable or not. Therefore, to judge the relative position of an object in the evolutionary ladder, vibration property could be used as a unique but cosmic trait, given that it is not merely understood in a narrow way. Some of the current literature claims that the use of four-dimensional printing or material is misleading, since 4D is not ordinarily perceivable by human perceptual senses. What these authors termed as 4D generally means a 3D product with timely changeable properties. However, this is not 4D, which indeed means ‘time’ is modifiable ‘back-forth’ as what can be done in other three dimensions [3,21,22]. Thus, 4D scaffold remains a long-future conceptual product, let alone a higher dimensional product beyond this. To term scaffold No.6 and 7 with possibly higher-dimensional properties could only be a theoretical indication, while not the decisive conclusion. To summarize, after the previous description regarding the seven-layered scaffold classification, Table 1 gives a brief summarization of the categorization work as communicated. 

### 3.4. Discussion on Futher Sub-Classification of Scaffold Under the Seven-Fold 

In previous sections, the seven-layer classification of scaffold has been brought up. It is logical to question the further sub-classes inside each of these seven scaffolds when scaffold is identified into one of them. A further classification inside each layer may help researchers better understand scaffolds. Work such as this will be meaningful when the seven-layer scaffold classification has become more mature and accepted. However, the basic point will still revolve around the idea of ‘evolution’, whereas ‘relativity’ will be based on ‘traits’ that can help scaffolds identical from one another. 

In an ordinary sense, scaffold classification is done according to external traits that can be partial or unidentical from the different experience or respective researcher point of view. In further developed engineering, which we could temporarily term it as ‘exact’ engineering, we assume that classification can be made based on ‘cosmic’ traits. As a matter of fact, there could exist some ‘exact’ traits which can be identical for every scaffold inside each of the seven categories, allowing future researchers to establish the finer sub-class of a given scaffold with possibly the utmost exactitude, both in relation to other scaffolds as well as to its own position in the ladder of scaffold’s evolutional process. From a current understanding, we suppose the ‘cosmic’ level of being of every scaffold could be determined via four aspects: first, by what this scaffold is made, produced, or fabricated; second, by what the scaffold ‘breathes’, i.e., how scaffold transmits air, nutrients, or other materials between vitro and vivo at an in-out process; third, by the cell culturing medium where the scaffold ‘lives’; and fourth, what functionality the scaffold serves cells. 

In brief, future work is needed to invest in the sub-classifying philosophy and prove its efficacy. Despite the potential usefulness of the new categorizing method, it still needs to cooperate with existing classifying methods, at least in its current stage. In other word, the new and traditional methods are not mutually exclusive and could work together. Therefore, other proven scaffold-categorizing methods, such as categorizing via natural or synthetic materials, 3DP or traditional fabricating method, scaffold-based or scaffold-free, 3D solid construct or hydrogel, being templates or permanently useable, types of decellularized matrix from various tissues and organs, and so forth, still have significant value as far as scaffold categorization is concerned. These could possibly be restructured and integrated inside the evolution-based scaffold classifying system, hopefully making the general scaffold categorization work more comprehensive, effective, and specific. 

## 4. Conclusions

This paper introduced the basic knowledge of cell culture scaffold, explaining the evolution line from static cell culture to dynamic cell culture, and from 2D cell culture to 3D. As scaffold engineering ascends into the cross-domain and miscellaneous, current mainstream classification of scaffold—for instance ‘traditional’ or ‘novel’—needs to be more specific, scientific, and persuasive. A new categorization method for scaffold engineering is therefore necessary, in order to avoid misunderstanding between researchers, as well as to increase the working efficiency inside scaffold studies. In this connection, the novel categorization of scaffold from No. 1, 2, 3, and into 7 has been established, with a summarizing table concluded the state-of-the-art of seven-layer scaffold classification via the language of ‘relativity’. 

From another point of view, an exact understanding of the language is necessary. In this paper, efforts have been made to define scaffold based on ‘relativity’, which could make it possible at once to mention what scaffold is being discussed, from what point of view, and in what connection. The fundamental property of the new definition is that all concepts of ‘scaffolds’, as well as their mutual relationship, are concentrated around one idea. This idea is about a scaffold’s ‘evolution’. Therefore, seven-levels of scaffolds have been defined to potentially illustrate the whole evolution process of scaffold engineering that covers the past, present, and future. With the betterment in scaffold’s categorization, future scaffold researchers might possibly be benefited, however unobvious, with increased clarity, precision, and understanding. 

In terms of future works, researchers may choose to focus on several aspects. First is to further analyse the classification of the seven layers and its philosophy. Since the novel language is herein introduced from the philosophical realm into scaffold engineering, there may be some comprehension difficulties, however small, which can be further modified and improved. Secondly, investigating the sub-classifying inside each of the seven categorizations, which was pointed out at paper’s end section, might also prove to be an interesting study. Solid sub-classifying could help the evolutional picture of scaffold engineering be more thorough. In addition, other works may involve introducing novel language of ’relativity’ into other engineering realms beyond the scaffold, which might in turn contribute to novel classification art that is better and more scientific. 

## Figures and Tables

**Table 1:** Summarizing table of scaffold categorization via introducing the language of ’relativity’.

Time Aspects	Previously Focused Cell Culture Scaffold			Bridging Scaffold	Short-Future Scaffold	Long-Future Scaffold	
Categorization	Scaffold No. 1	Scaffold No. 2	Scaffold No. 3	Scaffold No. 4	Scaffold No. 5	Scaffold No. 6	Scaffold No. 7
Dimensionality	2D	2.5D–3D	3D	3D	3D	3D or beyond	3D or beyond
Dynamicity	Static, Passive	Static, Passive	Static, Passive	Partly Dynamic, Active	Finely dynamic, Vibratory, Active	Highly Dynamic, Vibratory, Active	Ideally Dynamic, Vibratory, Active
Chief Feature in brief	2D plate or scaffold for 2D Cell Culture; very limited cell application; mostly replaced by scaffold No. 2 and 3; passive or static; stays at the bottom of scaffold’s evolutional ladder	3D Scaffold in early stage, simple characterization and single function, static or passive	Current mainstream 3D scaffold being passive or static, with increasingly tailored Characterizations on geometrics and composition	Recent scaffold partly made or designed by smart or dynamic materials; Bridging scaffold No. 1, 2, 3 to No. 5; potentially dynamic or active; inevitable stage toward achieving scaffold No. 5	3D scaffold with fully integrated vibratory functions; tailored- or self-vibratory properties; 3DPVS as one typical d; currently under conceptual development stage	Bridging scaffold No. 5 to No. 7; remains as a concept; Multiple-dynamic functions integrated inside scaffold might appear; much sophisticated properties	Ideal Scaffold in self-perception; Fully, ideally controllable vibratility; probably close the gap between in vitro and vivo
Evolutional Ladder	Relatively Lowest	Low	Moderate	Slightly High	High	Very High	Relatively Highest
